# Beer fermentation performance and sugar uptake of *Saccharomycopsis fibuligera*–A novel option for low-alcohol beer

**DOI:** 10.3389/fmicb.2022.1011155

**Published:** 2022-10-05

**Authors:** Yvonne Methner, Frederico Magalhães, Luis Raihofer, Martin Zarnkow, Fritz Jacob, Mathias Hutzler

**Affiliations:** ^1^Research Center Weihenstephan for Brewing and Food Quality, Technical University of Munich, Freising, Germany; ^2^Chair of Brewing and Beverage Technology, Technical University of Berlin, Berlin, Germany; ^3^VTT Technical Research Centre of Finland Ltd., Espoo, Finland

**Keywords:** *Saccharomycopsis fibuligera*, non-*Saccharomyces* yeasts, fermentation, brewing, low-alcohol beer, sugar uptake, micromanipulation, flavor

## Abstract

There is a growing trend for beers with novel flavor profiles, as consumers demand a more diversified product range. Such beers can be produced by using non-*Saccharomyces* yeasts. The yeast species *Saccharomycopsis fibuligera* is known to produce exceptionally pleasant plum and berry flavors during brewer’s wort fermentation while its mycelia growth is most likely a technological challenge in industrial-scale brewing. To better understand and optimize the physiological properties of this yeast species during the brewing process, maltose and maltotriose uptake activity trials were performed. These revealed the existence of active transmembrane transporters for maltose in addition to the known extracellular amylase system. Furthermore, a single cell isolate of *S. fibuligera* was cultured, which showed significantly less mycelial growth during propagation and fermentation compared to the mother culture and would therefore be much more suitable for application on an industrial scale due to its better flocculation and clarification properties. Genetic differences between the two cultures could not be detected in a (GTG)_5_ rep-PCR fingerprint and there was hardly any difference in the fermentation process, sugar utilization and flavor profiles of the beers. Accordingly, the characteristic plum and berry flavor could also be perceived by using the culture from the single cell isolate, which was complemented by a dried fruit flavor. A fermentation temperature of 20°C at an original gravity of 10 °P proved to be optimal for producing a low-alcohol beer at around 0.8% (*v*/*v*) by applying the *S. fibuligera* yeast culture from the single cell isolate.

## Introduction

The yeast species *Saccharomycopsis fibuligera* (synonym: *Endomyces fibuliger*) is known from food fermentations, especially from liqueur production (Aidoo et al., 2006; Xie et al., 2021). It was isolated from traditional fermented foods, such as Indonesian tapé made from glutinous rice or cassava tuber (Djien, 1972). In Southeast Asia in particular, the yeast species is used in rice wine fermentation and is known to produce desirable floral, fruity, and honey-like flavors during fermentation as a result of high production of esters and higher alcohols (Xie et al., 2021; Yang et al., 2021). Characteristic fruity, flowery, and honey flavors were also noted when *S. fibuligera* was cultured in selective media containing different carbohydrate sources (Lee et al., 2018). Due to its positive flavor properties, it was also tested for its suitability to ferment brewer’s wort, where there is a demand for diversification of the product range. As a result, *S. fibuligera* produced exceptionally promising plum-like, and also berry-like flavors in the beers (Srithamma, 2009; Methner et al., 2019, 2022). It was noticeable that, depending on the yeast strain, different sugar utilization patterns took place in brewer’s wort, so it was possible to produce both non-alcoholic and alcoholic beers. Lindner, who isolated the yeast species in the context of bread spoilage in 1907, described it as strongly utilizing sucrose during fermentation, weakly utilizing glucose, and unable to utilize maltose (Lindner, 1907). In contrast, Kreger-van-Rij stated in 1984 that the yeast species is capable of fermenting glucose as well as sucrose and maltose (Kreger-Van Rij, 1984). Nevertheless, existing literature explains that maltose utilization is slow (Kurtzman and Smith, 2011). Generally, *S. fibuligera* is known to possess amylolytic activity and therefore has the ability to degrade starch. In 1944, Wickerham observed the presence of an extracellular amylase system for *S. fibuligera* (Wickerham et al., 1944). Through several studies, the two enzymes α-amylase and glucoamylase from *S. fibuligera* were isolated and characterized (Futatsugi et al., 1993; Chen et al., 2010). The endoamylase α-amylase is capable of randomly cleaving α-1,4 glycosidic bonds and thus reducing amylose to oligosaccharides such as maltotriose and maltose. Glucoamylase (synonym: amyloglucosidase) yields individual glucose units from the hydrolysis of non-reducing ends of amylose and amylopectin and additionally cleaves maltose and maltotriose (Janeček and Baláž, 1992; Pandey, 1995; Tiwari et al., 2015). Hostinová observed that *S. fibuligera* can synthesize either both enzymes or only one type of amylase in a strain-specific manner (Hostinová, 2002). In a study by Methner et al. the glucoamylase activity gave an indication of why maltose from selective media could be fully metabolized by the yeast strain *S. fibuligera* S. fib Lu27 although hardly any maltose and maltotriose could be utilized by this strain from brewer’s wort (Methner et al., 2022). In contrast, the *S. fibuligera* yeast strain S. fib SF4 was able to partially metabolize maltose and maltotriose from brewer’s wort in another study (Methner et al., 2019). To date, it has not yet been investigated whether this yeast species utilized maltose due to extracellular amylase activity and subsequent passive glucose transport into the cell or due to transmembrane transporters (permeases) which actively transport maltose and/or maltotriose into the cell. Since the fermentation trials with S. fib Lu27 and S. fib SF4 were performed at different temperatures in the two aforementioned studies by Methner et al., a possible temperature dependence on the sugar utilization needs to be taken into consideration.

A distinctive characteristic of the yeast species *S. fibuligera* is its morphology, as it is dimorphic and forms individual round or oval budding cells as well as mycelia (Nga et al., 1995; Xie et al., 2021). The mycelium represents a challenge in terms of its suitability for brewing, since it does not flocculate, triggering a restriction of clarification of the beer. For this reason, single cells were isolated and recultivated in this study to establish in a direct comparison with the mother culture, whether the single cell isolation would influence the morphology during propagation and fermentation. To ensure that the culture of the isolated single cell had no genetic differences from the mother culture, additional (GTG)_5_ rep-PCR fingerprints were applied. Furthermore, since the yeast should retain the ability to produce exceptionally fruity flavors during the fermentation of brewer’s wort as a consequence of the single cell isolation, the sensory properties of the final beers were studied.

The objective of this study was to elucidate whether the yeast species *S. fibuligera* possesses an active transport system for maltose and maltotriose, which was examined by maltose and maltotriose transport assays. Furthermore, it was investigated – exemplified by the yeast strain S. fib SF4 – whether it was possible to reduce the characteristic mycelial formation by means of micromanipulation and recultivation in order to increase the suitability for brewing on an industrial scale. Possible influences of micromanipulation on brewing potential including sugar utilization and beer flavor profiles were studied and compared with the mother culture and the domesticated reference lager yeast strain *Saccharomyces pastorianus* TUM 34/70 at fermentation temperatures of 20 and 28°C.

## Materials and methods

### Yeast strains

Table 1 lists the yeast strains with the corresponding abbreviations that were investigated or used as reference yeast strains in this study. While the two *S. fibuligera* yeast strains represented the yeasts under investigation, *S. pastorianus* S. pas 34/70 served as the reference yeast strain for all conducted experiments. *S. eubayanus* S. eub 12357 and *S. ludwigii* S. lud SL17 were used as additional reference yeasts for the maltose and maltotriose transport assays.

**Table 1:** Yeast species and strain numbers with corresponding abbreviations used in this study.

Yeast strain number	Yeast strain abbreviation	Yeast species
TUM SL17	S. lud SL17	*Saccharomycodes ludwigii*
VTT C-12902/CBS12357	S. eub 12357	*Saccharomyces eubayanus*
VTT A-13220/TUM 34/70	S. pas 34/70	*Saccharomyces pastorianus*
PI S 6; Lu27	S. fib Lu27	*Saccharomycopsis fibuligera*
PI S 7; Lu 26/SF4	S. fib SF4	*Saccharomycopsis fibuligera*

### Maltose and maltotriose uptake activity

S. lud SL17 was used as a negative control as the yeast strain is unable to take up maltose or maltotriose during fermentation (Boundy-Mills et al., 2011) whereas S. eub 12357 is known to be maltose-positive but maltotriose-negative during fermentation and thus represented the maltotriose-negative control (Gibson et al., 2013). S. pas 34/70, which represents a traditional group II lager yeast can utilize both maltose and maltotriose and was therefore used as the positive control. The maltose and maltotriose uptake activity of the two yeast strains S. fib Lu27 and S. fib SF4 were to be determined. For maltose and maltotriose uptake measurement, S. lud SL17 was grown in YP medium prepared from 1.0% yeast extract (Sigma-Aldrich, St. Louis, MO, U.S.A.), 2.0% peptone from casein, pancreatic digest (Sigma-Aldrich, St. Louis, MO, U.S.A.) and 1.0% glucose. S. eub 12357 was grown in YP containing 1.0% maltose and the remaining three strains (S. pas 34/70 and the two *S. fibuligera* S. fib Lu27 and S. fib SF4) were grown in YP medium containing maltose (1% *w*/*v*) or maltotriose (1% *w*/*v*). All strains were propagated at 20°C in liquid medium to an OD_600 nm_ between 4 and 8. The cells were harvested by centrifugation (10 min, 2,968 g, 0°C), washed twice with ice-cold water and once with 0.1 M tartrate-Tris (pH 4.2) before being re-suspended in the same buffer to a concentration of 200 mg/ml fresh yeast. Zero-trans rates of [U-^14^C]-maltotriose uptake were measured at 20°C essentially as described by Lucero et al. (1997). Briefly, aliquots of 40 μl of yeast suspension were added to 20 μl of 15 mM labeled maltotriose (for a final concentration of 5 mM [U-^14^C]-maltotriose) and incubated for 60 s at 20°C. The reaction was stopped by adding 5 ml ice-cold water. The suspension was immediately filtered and washed with an additional 5 ml ice-cold water. The filter was submerged in 3.5 ml of Optiphase HiSafe 3 scintillation cocktail (Perkin Elmer, MA, United States) and the radioactivity measured in a Perkin Elmer Tri-carb 2,810 TR scintillation counter. [U-^14^C]-maltotriose (ARC 627) was obtained from American Radiolabeled Chemicals (St. Louis, MO, United States) and re-purified before use as described by Dietvorst et al. (2005). Maltose (minimum purity, 99%) and maltotriose (minimum purity, 95%) were from Sigma-Aldrich (St. Louis, MO). The cell washing was expected to remove any potential extracellular carbohydrate-hydrolase that could interfere with the results. In addition, keeping cells on ice until the uptake assay, the short incubation time in maltotriose, and subpar temperature and pH conditions for known extracellular glucoamylases of the *Saccharomyces* genus (pH*_Opt_* = 4.5–6, T*_Opt_* = 40–60°C; (Hostinová and Gašperík, 2010)) were expected to limit the activity of any residual carbohydrate hydrolase that might be present.

### Micromanipulation and yeast propagation

For micromanipulation, a TransferMan® 4r micromanipulator with DualSpeed™ Joystic and CellTram® 4r Air, a pneumatic manual microinjector, were used (Eppendorf SE, Hamburg, Germany). The capillary with an inner diameter of 10 μm (BioMedical Instruments, Zöllnitz, Germany) was connected to a Nikon eclipse T*i-e* inverse microscope (Nikon, Tokyo, Japan) using an adapter. For micromanipulation, 3 ml of sterilized wort (100°C, 45 min) with an original gravity of 10 °P was pipetted into three sterile cell culture dishes (35 × 10 mm; Greiner Bio-One GmbH, Frickenhausen, Germany). Wort was prepared from unhopped malt extract (Weyermann®, Bamberg, Germany) by re-dilution with distilled water. The yeast strain S. fib SF4 was transferred from a wort slant agar (mother culture) into the wort of one cell culture dish and was distributed using a sterile inoculation loop. Using the micromanipulator, two single cells of the yeast strain S. fib SF4 were isolated and one cell per cell culture dish was transferred into the wort. Due to the filamentous growth of *S. fibuligera*, care was taken not to isolate filaments from the mother culture along with the single cells. One cell culture dish was incubated at 20°C for 72 h, the other sample was incubated at 28°C for 72 h. Temperatures at 20 and 28°C were selected, since there are already existing studies with *S. fibuligera* yeast strains in brewer’s wort on these two approximate temperatures (Methner et al., 2019, 2022). The yeast cultures grown in the cell culture dishes were each transferred to 50 ml sterile wort in 100 ml flasks sealed with cotton plugs and propagated for another 72 h at the respective temperatures on a WiseShake orbital shaker (Witeg Labortechnik GmbH, Wertheim, Germany) at 80 rpm. Simultaneously, S. fib SF4 mother culture from wort slant agar was incubated under sterile conditions into 2 × 50 ml of the identical wort in 100 ml flasks. Here as well, one sample was propagated at 20°C, while the second sample was propagated at 28°C. At the end of the 72 h propagation, 1 ml material of the four different samples was removed in each case into sterile 1.5 ml SafeSeal micro tubes (Sarstedt AG & Co. KG, Nümbrecht, Germany) for (GTG)_5_ rep-PCR fingerprint analysis. The remaining propagation yeasts were transferred to 250 ml fresh, sterile 10 °P wort in 500 ml flasks and propagated for an additional 72 h at the respective parameters selected at the outset. In a final propagation step, the four samples were transferred to 1800 ml sterile wort in 2500 ml flasks and propagated for another 72 h before the yeasts were further processed for fermentation. Since a reference beer was to be produced for the fermentations in addition to the four experimental beers, the yeast strain S. pas 34/70 was also propagated as described as a commercial domesticated lager yeast strain for brewing. Additionally, comparative microscopic images were taken of the mother culture S. fib SF4 from the wort slant agar and the two yeasts propagated at 28°C (mother culture and micromanipulated culture) to compare the morphologies using the aforementioned brightfield Nikon inverse microscope at a magnification of × 40 (objective) plus digital zoom (scale is visible in microscopic pictures).

### (GTG)_5_ rep-PCR fingerprint

(GTG)_5_ rep-PCR fingerprint system was applied to determine if the four differently propagated *S. fibuligera* S. fib SF4 yeast cultures had identical or different genetic fingerprints. According to Versalovic et al. (1994), the primer (GTG)_5_ (5′-GTG GTG GTG GTG GTG-3′) was originally developed for bacteria and was successfully transferred to differentiate various non-*Saccharomyces* yeasts (Meyer et al., 1993; Erdem et al., 2016). After sample preparation, DNA fingerprint amplification was performed, followed by capillary gel electrophoresis and the data processing of the generated fingerprints. Table 2 lists the four different propagation approaches of the *S. fibuligera* yeast strain S. fib SF4 with their corresponding varying parameters and abbreviations, which were subsequently investigated.

**Table 2:** Four different propagation approaches with the corresponding varying parameters of the yeast strain *Saccharomycopsis fibuligera* SF4.

Abbreviation	Yeast culture	Propagation and fermentation temperature
SF4-MI-20	Micromanipulated	20°C
SF4-MK-20	Mother culture	20°C
SF4-MI-28	Micromanipulated	28°C
SF4-MK-28	Mother culture	28°C

For sample preparation, DNA was first isolated from the liquid samples. For this purpose, the yeast-wort suspensions were centrifuged (Mikro 200, Andreas Hettich GmbH & Co. KG, Tuttlingen, Germany) in sterile 1.5 ml SafeSeal microtubes (Sarstedt AG & Co. KG, Nümbrecht, Germany) at 16,000 g for 2 min and the supernatant was discarded. 200 μl Insta-Gene Matrix was added to the samples before they were incubated in the thermomixer preheated to 56°C for 30 min. Samples were then vortexed in the tubes and placed in the 95°C preheated thermomixer for an additional 8 min. After a further centrifugation step at 16,000 g for 2 min, 100 μl of the supernatant was transferred to fresh sterile tubes. In the next step, DNA concentrations were measured using NanoDrop1000 spectrophotometer (Peqlab Biotechnologie GmbH, Erlangen, Germany) and adjusted to 25 ng/μL, containing 12.5 μl RedTaq Mastermix (2×) 2-fold (Genaxxon bioscience, Ulm, Germany), 10 μl Primer Solution (50 pmol/l), 5 μl PCR-clean double distilled water (ddH_2_O) and 2.5 μl sample DNA. The PCR temperature protocol of the DNA fingerprint amplification and subsequent capillary gel electrophoresis including data processing were described according to Riedl et al. (2019).

### Fermentation and beer analysis

Before starting the small-scale fermentation trials in triplicate, the yeast cell counts for the reference strain S. pas 34/70 and the four individually propagated S. fib SF4 cultures were determined. The Cellometer® Vision (Nexcelom Bioscience LLC, Lawrence, MA, United States) was used to determine cell counts. The pitching rate for the fermentation experiments was set at 10 × 10^6^ cells/mL (± = 1 × 10^6^ cells/mL). After cell counting, the corresponding calculated propagation yeast volumes were centrifuged (Roto Super 40, Andreas Hettich GmbH & Co. KG, Tuttlingen, Germany) at 750 g for 5 min in sterilized 500 ml PPCO centrifuge bottles (Nalgene, Thermo Fisher Scientific, Waltham, MA, USA). Subsequently, the wort supernatant was discarded and the yeast samples were pitched into 1800 ml unhopped sterilized wort (10.2 °P, pH 5.3) prepared from malt extract (Weyermann®, Bamberg, Germany) in 2000 ml sterile Duran glass bottles (Schott AG, Mainz, Germany). The fermentation bottles were closed with glass fermentation airlocks on top. While yeast cultures already propagated at 28°C were also fermented at 28°C, yeast cultures propagated at 20°C were fermented at 20°C accordingly. By weighing the samples every 24 h, the fermentation progress was monitored by weight loss, which was mainly due to escaping carbon dioxide and based on Balling’s assumption that during fermentation, an average of 2.0665 g of extract is converted into 1 g alcohol, 0.9565 g carbon dioxide and 0.11 g yeast (Esslinger, 2009). Following the main fermentation, which lasted for 480 h (20 days), the samples were sealed with sterile screw caps and cooled to 2°C for an additional 168 h (7 days) before the analyses shown in Table 3 were performed. For the sugar utilization results, one-sample *t*-tests were performed using OriginPro 2020 as statistical software to evaluate if the mean values of carbohydrate utilizations of the different fermentations varied significantly.

**Table 3:** Analytical methods of the wort and the beers according to MEBAK^a^ and Donhauser et al.^b^.

Analysis	Method	Device
Original gravity, apparent attenuation, ethanol content	MEBAK^a^ WBBM 2.9.6.3	Bending vibration and NIR spectroscopy, Alcolyzer Plus with DMA 5000 X sample 122 (Anton-Paar GmbH, Ostfildern, Germany)
pH value	MEBAK^a^ WBBM 2.13	pH meter with pH electrode, ProfiLine pH3210 pH meter (Xylem Inc., New York, NY, United States)
Sugar composition (glucose, fructose, sucrose, maltose, maltotriose)	Donhauser et al.^b^ LS-HPLC 002_2	HPLC UltiMate 3000 (Thermo Fisher Scientific, Waltham, MA, United States)

a MEBAK® (2012), Editor: Dr. F. Jacob: The MEBAK collection of brewing analysis methods: Wort, beer and beer-based beverages. Collection of methods of the Mitteleuropäischen Brautechnischen Analysenkommission. Self-published by MEBAK.

b Donhauser, S.; Wagner, D. (1990): Zucker- und Endvergärungsgradbestimmung mittels der HPLC, 9:306–309. Monatsschrift für Brauwissenschaft.

### Sensory evaluation

The beer samples were tempered to 12°C and were profiled at 20°C room temperature by a sensory panel of eight DLG (Deutsche Landwirtschafts-Gesellschaft e.V., Frankfurt, Germany)-certified assessors. The accredited sensory evaluations were conducted according to DIN EN 17025. To exclude external interferences during the tastings, they were held in an appropriately neutral, white-colored room with individual tasting chambers. A sensory test was carried out according to the DLG evaluation scheme, which comprises a rating scale from zero to five. While zero is considered the lowest score and represents insufficient product quality, a score of five points fully meets the quality expectations of the product and matches the quality description very well (Hildebrandt and Schneider-Haeder, 2009). The odor, purity of taste and body of the beers were evaluated, while the quality of bitterness as well as carbonation were neglected as the beers were produced from unhopped malt extract and the fermentations were conducted without pressure. For the tasting, the samples were assigned randomized three-digit numbers and 50 ml of each sample was served in brown 200 ml tasting glasses. There was also an additional sensory test method – a descriptive tasting. The method was based on the descriptive sensory evaluation of Meier-Dörnberg et al. (2017) with the seven main categories of tropical fruity, fruity (other fruits), citrus, spicy, floral, malty, and other flavors. The sensory assessors were asked separately about beer odor and taste, which they scored from 0 (not noticeable) to 5 (extremely noticeable). The results were statistically analyzed by first determining the interquartile ranges and removing extreme outliers (3 × IQR) to obtain only statistically significant values. For significant results, the mean values of odor and taste were calculated before the beer samples fermented with the *S. fibuligera* yeast strain S. fib SF4 were compared with the reference beers fermented with *S. pastorianus* S. pas 34/70.

## Results

### Maltose and maltotriose uptake activity

**Figure 1:**
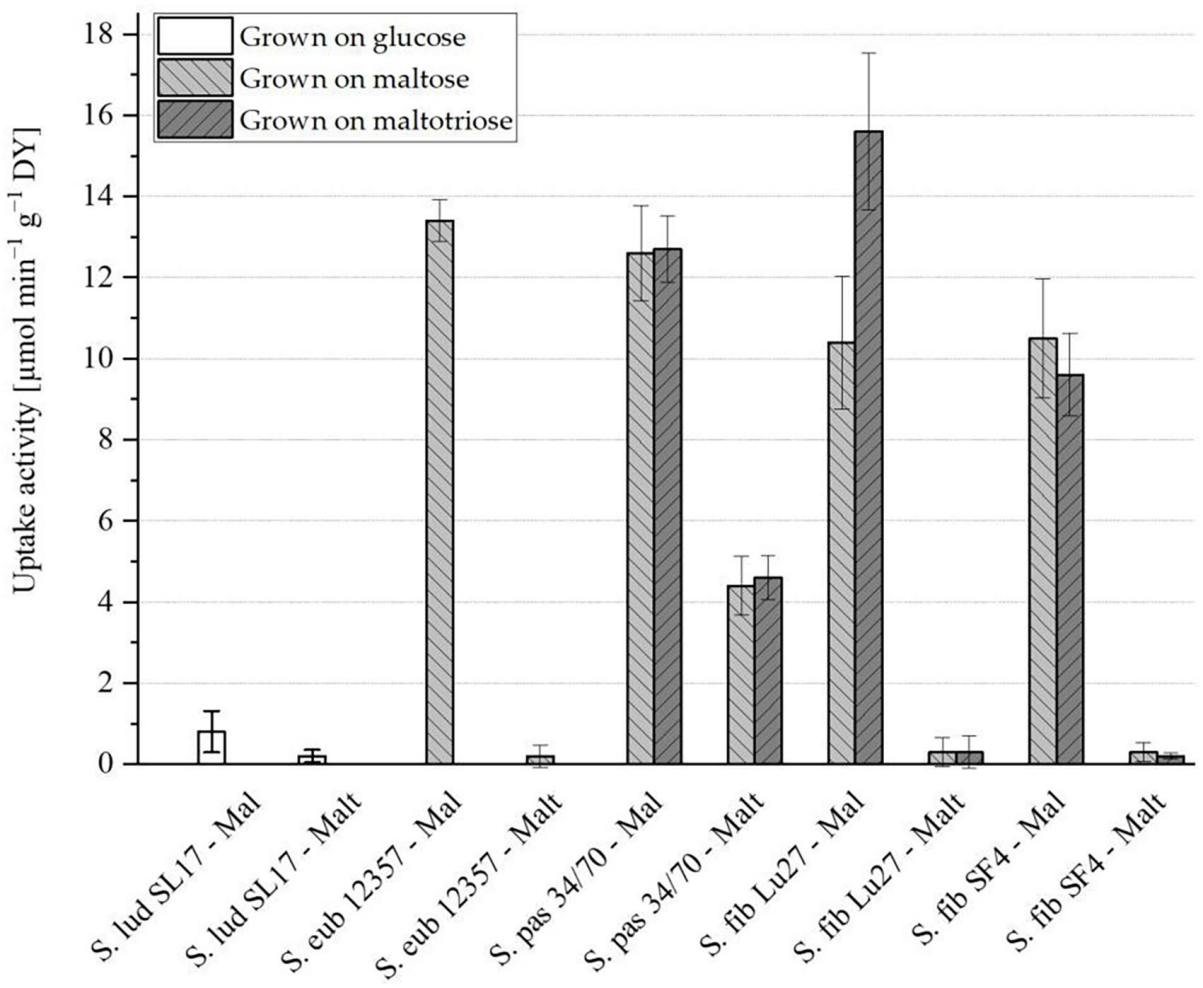
Zero-trans rates of maltose (Mal) and maltotriose (Malt) uptake activity (μmol min^−1^ g^−1^ dry yeast (DY)) measured at 20°C for cells propagated on glucose (white), maltose (light grey) or maltotriose (dark grey) with *n* = 4. An uptake activity equal or below 0.5 μmol min^−1^ g^−1^ DY is considered negligible. Yeast strain abbreviations according to Table 1.

Prior to the uptake activity assessment, the strains were propagated when possible in both maltose and maltotriose as sole carbon sources. Due to the inability of S. eub 12357 to grow on maltotriose, it was propagated only on maltose. S. lud SL17 cannot grow on maltose nor maltotriose and was therefore only propagated in glucose. The results of the maltose and maltotriose uptake activity are presented in Figure 1, respectively. Raw data can be found in the Supplementary material Table 1.

*S. ludwigii* S. lud SL17 maltose uptake activity was just above the limit at 0.8 μmol min^−1^ g^−1^ DY but not significantly (standard deviation of ±0.51; *cf.* Figure 1). This strain is known to be maltose negative and the higher measured value is likely a result of experimental variability. Maltotriose activity was, however, clearly below the minimum activity required (cf. Figure 1). *S. eubayanus* S. eub 12357, as expected, showed high maltose uptake activity and no maltotriose uptake activity. The strain S. pas 34/70 had maltose uptake activity comparable to that of S. eub 12357 and it was the only strain with maltotriose uptake activity significantly above 0.5 μmol min^−1^ g^−1^ DY. Growth on maltose or maltotriose generally did not seem to affect the uptake activity of either sugar. The only exception was S. fib Lu27, which had 50% higher maltose uptake activity when grown on maltotriose. Otherwise, the *S. fibuligera* strains S. fib Lu27 and S. fib SF4 showed very similar behavior. Maltose uptake activity was lower but still comparable to that of S. eub 12357 and S. pas 34/70. Maltotriose activity was below 0.5 μmol min^−1^ g^−1^ DY for both strains irrespective of the sugar used. However, the error is quite high, particularly for S. fib Lu27. This can partly be explained by the difficulty of homogenizing cells with filamentous growth. This created challenges for the uptake experiments and the measurement of cell mass. Regardless, the results strongly suggest that *S. fibuligera* does not have any significantly active transporters able to take up maltotriose into the cells. Maltotriose utilization is therefore dependent on its extracellular hydrolysis followed by uptake of the resulting glucose or maltose molecules. Maltose, however, can be both hydrolyzed extracellularly or intracellularly due to active maltose transmembrane transporters. Due to the identical sugar transport systems of the two investigated *S. fibuligera* yeast strains, S. fib SF4 and S. fib Lu 27, and the higher fermentation activity of the yeast strain S. fib SF4 which was reported in two previous studies by Methner et al. (2019), the yeast strain S. fib SF4 was selected for all further trials in this study.

### Morphology of *Saccharomycopsis fibuligera*

**Figure 2:**
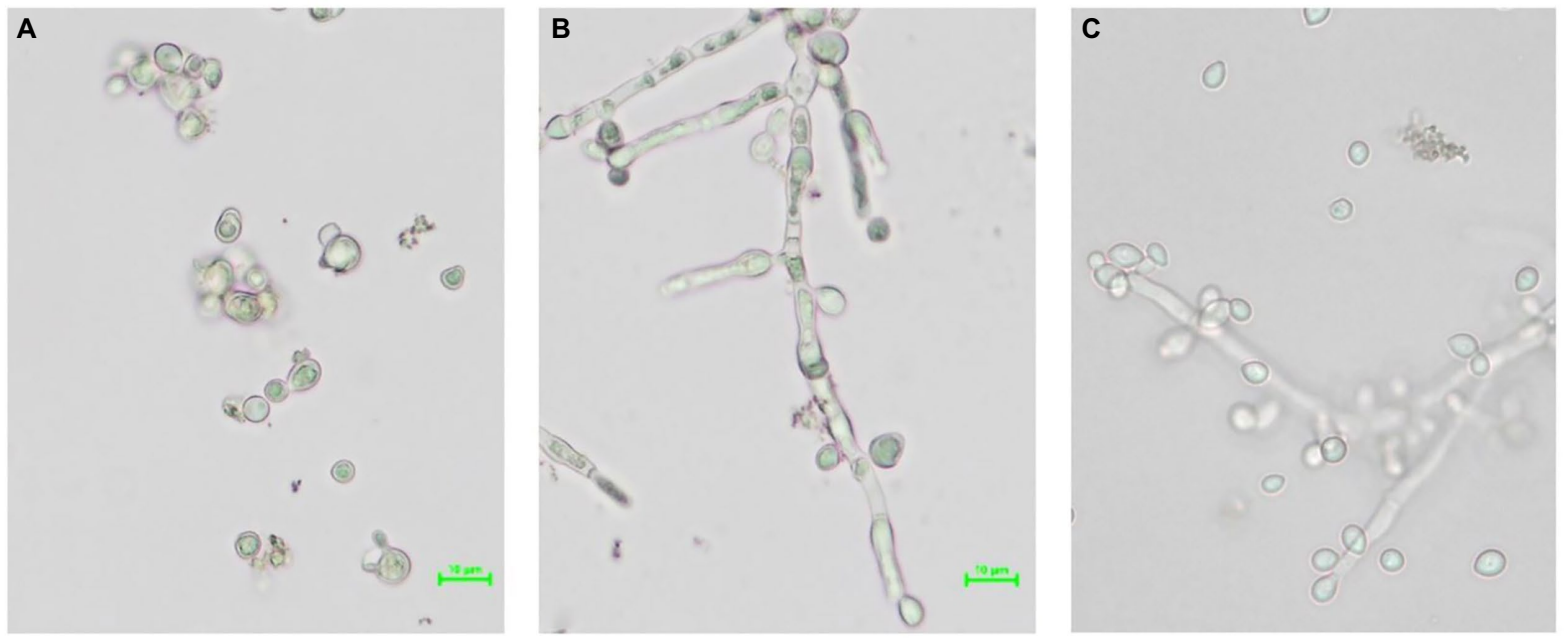
Brightfield microscopy pictures at a magnification of × 40 (objective) plus digital zoom [scale is visible in microscopic photos **(A)** and **(B)**] showing different morphologies of the yeast strain *S. fibuligera* S. fib SF4: **(A)** SF4-MI-28, **(B)** SF4-MK-28, **(C)** SF4 from slant agar.

A single cell without filaments of the yeast strain *S. fibuligera* S. fib SF4 was isolated and propagated in brewer’s wort as well as the mother culture S. fib SF4 as described in Section 2.3. Figure 2 depicts the comparison of the morphologies in a brightfield microscope at a magnification of × 40 (objective) plus digital zoom [scale is visible in microscopic pictures Figure 2A, B] during growth at 28°C of the micromanipulated yeast culture Figure 2A in comparison with the mother culture during growth at 28°C Figure 2B and the original culture from the wort slant agar before propagation Figure 2C.

Comparing the three microscopic images with each other, there are clear differences in morphology. While the micromanipulated yeast culture hardly formed filaments during growth at 28°C and showed budding cells (A), the mother culture predominantly developed mycelia at the same propagation temperature (B). The original culture from slant agar, which unlike the yeasts in (A) and (B) was not in log but in stationary phase, showed both mycelia and single cells in the microscopic image (C). At a propagation temperature of 20°C, the cell morphology of the three cultures A-C did not differ from the respective clone cultures propagated at 28°C. Therefore, these images are not shown.

### (GTG)_5_ rep-PCR fingerprint

**Figure 3:**
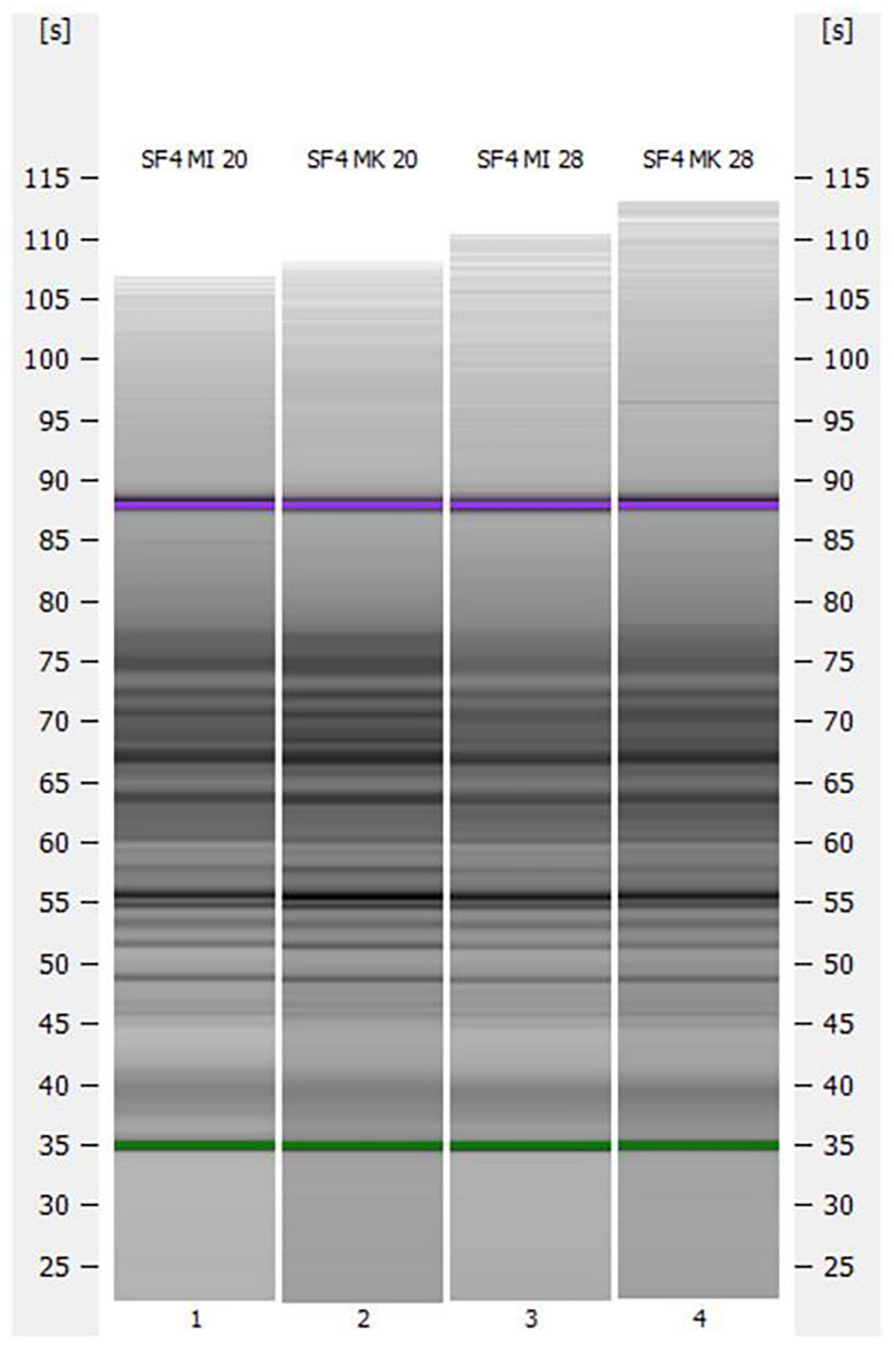
Capillary gel electrophoresis (GTG)_5_ patterns for the investigated yeast strain *S. fibuligera* S. fib SF4 micromanipulated and propagated at 20°C (SF4 MI 20) and 28°C (SF4 MI 28) and the mother culture propagated at 20°C (SF4 MK 20) and 28°C (SF4 MK 28).

Based on the different morphologies in Section 3.2, a (GTG)_5_ rep-PCR fingerprint was performed to investigate whether the two micromanipulated isolates were genetically different from the mother culture. Accordingly, the fingerprint analysis was carried out with both micromanipulated cultures propagated at 20°C and 28°C and with the mother culture propagated at 20°C and 28°C. Previous studies revealed that the (GTG)_5_ fingerprinting is not only a suitable method to differentiate all species of beer-spoiling bacteria, but also to differentiate non-*Saccharomyces* yeasts on strain level (Michel et al., 2016; Riedl et al., 2019). The results are depicted in Figure 3.

Although there were differences in morphology in the brightfield microscopy (*cf.* Section 3.2), no obvious differences could be detected in the (GTG)_5_ patterns of the capillary gel electrophoresis.

### Analytical results

**Figure 4:**
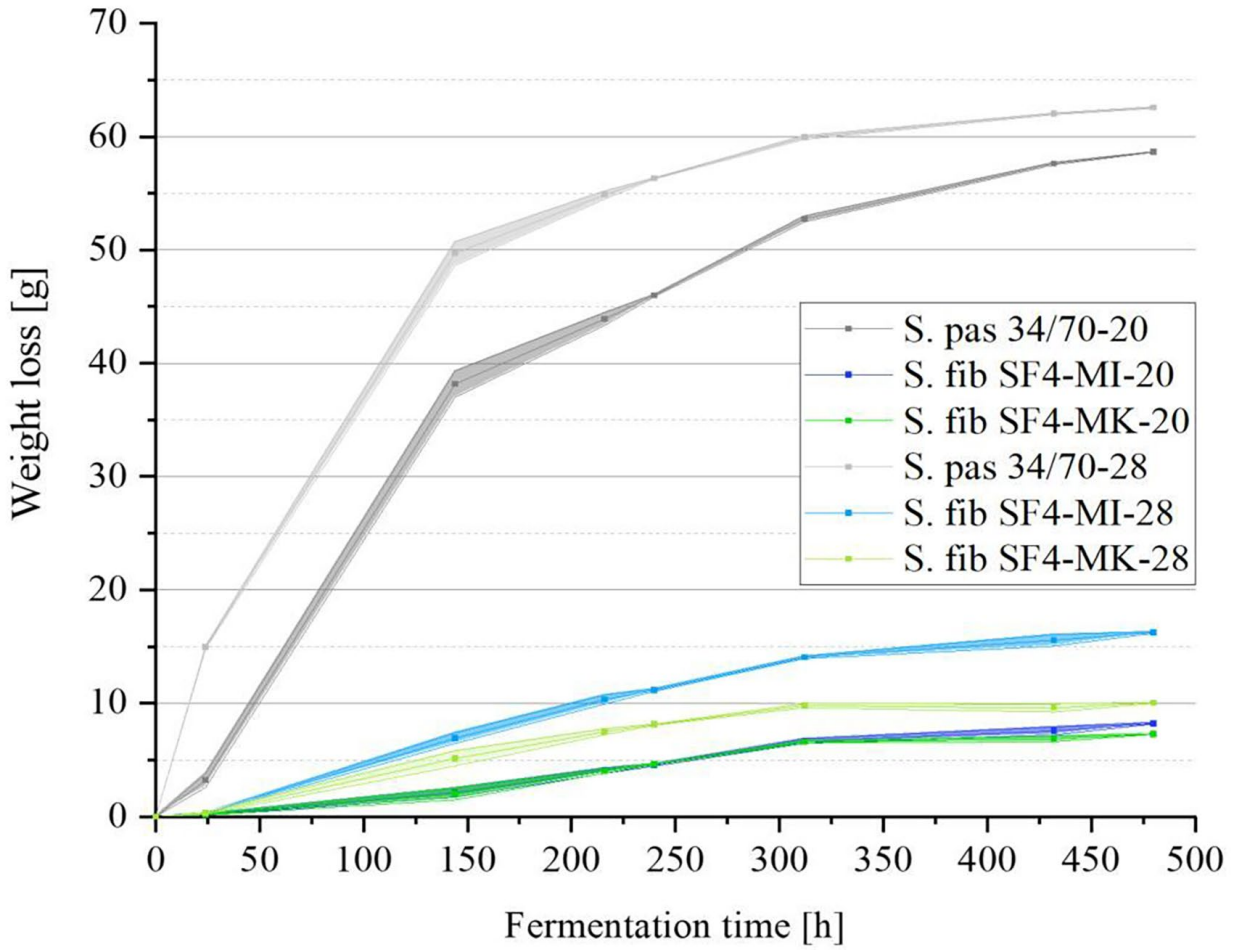
Mean values (*n* = 3) with standard deviations of the fermentation samples’ total weight losses in grams of the investigated yeast strain S. fib SF4 as micromanipulated (MI) and mother culture (MK) and the reference yeast strain S. pas 34/70 during the fermentation process over a fermentation period of 480 h (20 days) at 20 and 28°C.

To investigate the fermentation process, the weight losses of the six samples were checked every 24 h during fermentation and can be viewed in Figure 4. Raw data can be viewed in the Supplementary material Table 2. Due to the unpressurized fermentation, the carbon dioxide formed during fermentation was able to escape, resulting in a weight loss. The reference yeast strain S. pas 34/70 generally showed a significantly higher fermentation activity than the yeast S. fib SF4. While S. pas 34/70 already lost around 40 g in weight within the first six fermentation days (144 h) at a temperature of 20°C, the weight loss at 28°C was even higher at around 50 g. For the sample fermented at 20°C, this accounted for around two thirds of the final weight loss after 20 days, while the sample fermented at 28°C had already reached almost 80% of the final weight loss after only six days. In contrast, the S. fib SF4 fermentations lost significantly less weight than those from the reference strains. After 20 days, the sample S. fib SF4-MI-28 lost the most weight at about 16 g in direct comparison with the other S. fib SF4 samples. S. fib SF4-MI-20 only achieved about half of this amount, at approximately 8 g. Despite the higher fermentation temperature, S. fib SF4-MK-28 lost an insignificant amount of weight at around 10 g, and S. fib SF4 -MK-20 brought up the rear with just about 7 g weight loss after 20 days of fermentation.

The high weight losses of the two reference beers fermented with S. pas 34/70 at 20°C and 28°C correspond to the high apparent attenuations over 80% and ethanol concentrations of 4.5–4.6% (*v*/*v*), as can be seen in Table 4. Both apparent attenuation and ethanol concentration of the reference sample fermented at 28°C were slightly higher than the concentrations of the sample fermented at 20°C, which is consistent with the weight losses shown in Figure 4. Consequently, it was expected that the values for the S. fib SF4 samples would be significantly lower, which was in fact the case. The results are also listed in Table 4. Among the four S. fib SF4 beers, the beer fermented with the micromanipulated yeast culture at 28°C had comparatively the highest apparent attenuation at 23% and an ethanol content of 1.2% (*v*/*v*). The other three beers fermented with S. fib SF4, which showed similar weight losses during the fermentation process (*cf.* Figure 4), revealed comparable apparent attenuations (around 14–16%) and ethanol contents (0.74–0.83% (*v*/*v*)). Although the apparent attenuations of the beers fermented with the S. fib SF4 yeast cultures were low compared with the two reference beers, the pH drop was more pronounced. While the two reference beers had a pH of around 4.6, pH values of between 4.16–4.43 were measured in the S. fib SF4 beers. Strikingly, the two beers from the micromanipulated cultures were lower than the beers from the mother culture with pH values of 4.16 (SF4-MI-28) and 4.33 (SF4-MI-20). It cannot be explicitly explained and would need further research to establish why *S. fibuligera* caused a stronger pH drop in the beers than the reference yeast *S. pastorianus*. However, it is known that yeast cells acidify their environment during nutrient transport by a combination of direct secretion of organic acids, excretion of protons and CO_2_ dissolution (Budroni et al., 2017). These metabolic processes could be comparatively more pronounced in *S. fibuligera* yeast cells. A reduced buffer capacity could also be a potential reason for the low pH. In order to gain a deeper understanding of why the pH value was comparatively lower than in the two reference beers produced with the *Saccharomyces* yeast strain, organic acids could be measured in future investigations. This was not carried out as part of this study, as the question was not focused.

**Table 4:** Original gravity [%], apparent attenuation [%], ethanol content [% (*v*/*v*)], and pH values in the final beers fermented with the yeast strain *S. fibuligera* S. fib SF4 (mother culture and micromanipulated culture) and the reference yeast strain *S. pastorianus* S. pas 34/70 at fermentation temperatures of 20 and 28°C.

Yeast strain	Original gravity [°P]	Apparent attenuation [%]	Ethanol [% (*v*/*v*)]	pH value
S. pas 34/70-20	10.12 ± σ = 0.01	81.40 ± σ = 0.08	4.47 ± σ = 0.00	4.61 ± σ = 0.01
S. fib SF4-MI-20	10.17 ± σ = 0.01	15.83 ± σ = 0.17	0.83 ± σ = 0.01	4.33 ± σ = 0.01
S. fib SF4-MK-20	10.18 ± σ = 0.01	13.97 ± σ = 1.01	0.74 ± σ = 0.05	4.43 ± σ = 0.02
S. pas 34/70-28	10.13 ± σ = 0.01	82.20 ± σ = 0.08	4.57 ± σ = 0.01	4.58 ± σ = 0.00
S. fib SF4-MI-28	10.18 ± σ = 0.02	22.73 ± σ = 1.39	1.20 ± σ = 0.07	4.16 ± σ = 0.03
S. fib SF4-MK-28	10.18 ± σ = 0.00	15.77 ± σ = 0.92	0.83 ± σ = 0.05	4.38 ± σ = 0.02

**Figure 5:**
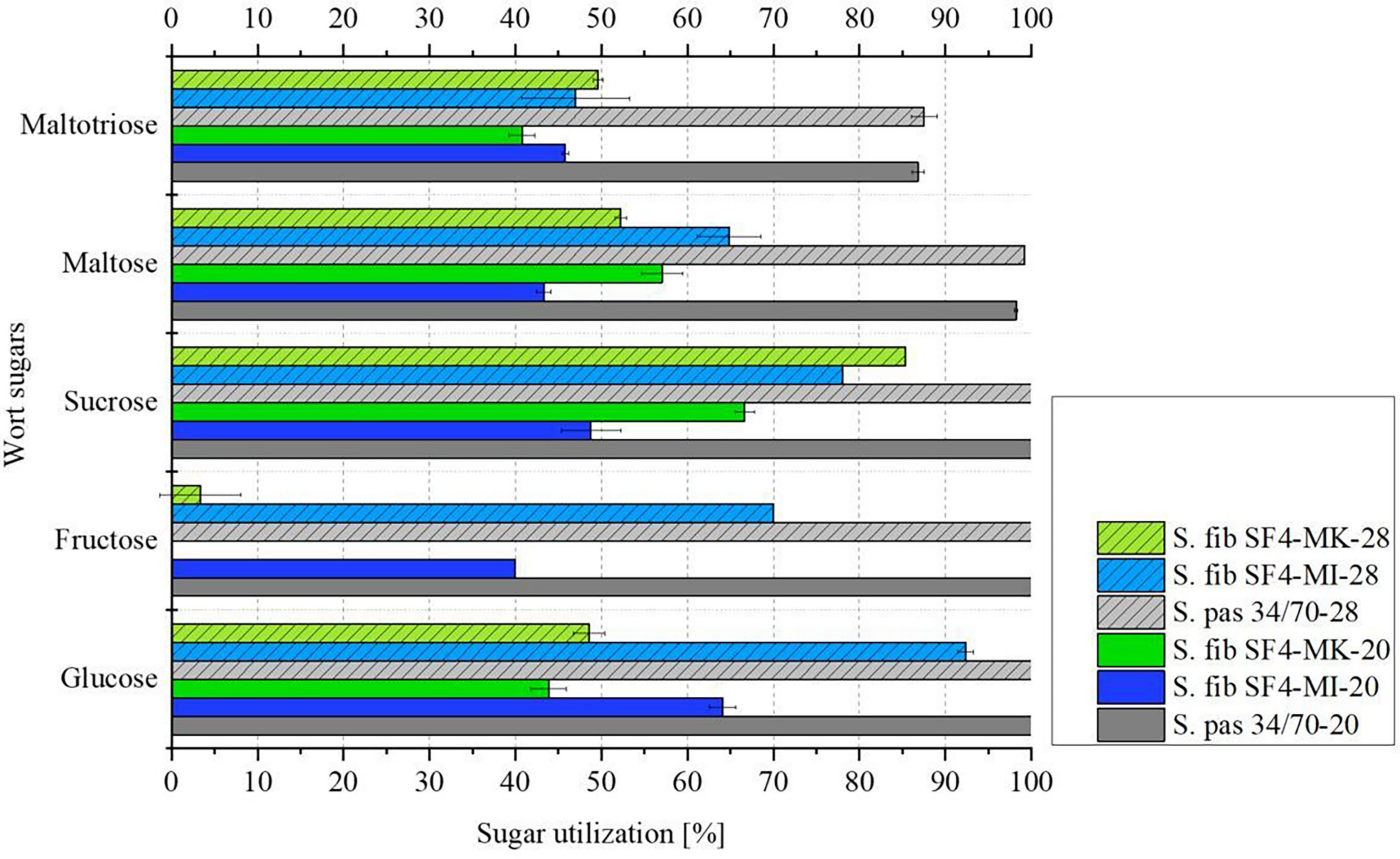
Mean values (*n* = 3) with standard deviations of wort sugar utilization in % of the investigated yeast strain S. fib SF4 as micromanipulated (MI) and mother culture (MK) and the reference yeast strain S. pas 34/70 during fermentation from wort to beers at 20 and 28°C.

Table 5 illustrates the sugar composition of the main wort carbohydrates glucose, fructose, sucrose, maltose and maltotriose (Narziß et al., 1999) used for the fermentation experiments. Both the wort sugar composition and the sugar composition of the beers were analyzed in order to draw conclusions about the sugar utilization of the individual yeast cultures during fermentation. Figure 5 depicts the sugar utilization by the individual yeast cultures in percent. Raw data can be found in Supplementary material Table 3.

**Table 5:** Sugar composition of the wort used for fermentation trials.

Wort sugar	Concentration in g/L
Glucose	9.2
Fructose	1.0
Sucrose	4.1
Maltose	51.0
Maltotriose	13.9

The sugar utilization of the yeasts also reflects that the reference yeast strain S. pas 34/70 was significantly more active than the experimental yeast S. fib SF4 at both 20°C and 28°C fermentation temperatures. One-sample *t*-tests were performed using OriginPro 2020 as statistical software to demonstrate that the mean values of the maltose and maltotriose utilization results were significantly different between the reference yeast strain S. pas 34/70 and the experimental yeast S. fib SF4 at both temperatures (*cf.* Supplementary material Table 4). The reference yeast completely utilized glucose, fructose and sucrose present in the wort, maltose at 98% (20°C) to 99% (28°C), and maltotriose at 87% (20°C) to 88% (28°C). In contrast, none of the wort sugars was fully utilized by the S. fib SF4 cultures during the fermentation time of 20 days. In the beer fermented with S. fib SF4-MI-28, which showed the highest fermentation activity in direct comparison with the other *S. fibuligera* cultures, the highest sugar utilization was measured. Glucose utilization was above 90% and on average not significantly different from the reference S. pas 34/70 at 28°C according to the one-sample *t*-test result (*cf.* Supplementary material Table 4). In general, the range of maltose utilization of the S. fib SF4 cultures was between 43 and 65%, for maltotriose it was between 41 and 50%. For glucose, fructose and sucrose, significantly larger ranges of variation were analyzed. Only sucrose utilization of S. fib SF4-MI-28 and S. fib SF4-MK-28 were not significantly different from each other, while all other results for glucose, fructose, and sucrose were significantly different regardless of the fermentation temperature (*cf.* Supplementary material Table 4). The two beers fermented with the S. fib SF4 mother culture stood out, as hardly any fructose was utilized and only about half of the glucose. The micromanipulated cultures showed higher utilization rates, but here, too, the wort sugars were only partially utilized by the yeasts. The incomplete sugar utilization explains the low apparent attenuations and the low ethanol concentrations from Table 4.

### Sensory evaluation

**Figure 6:**
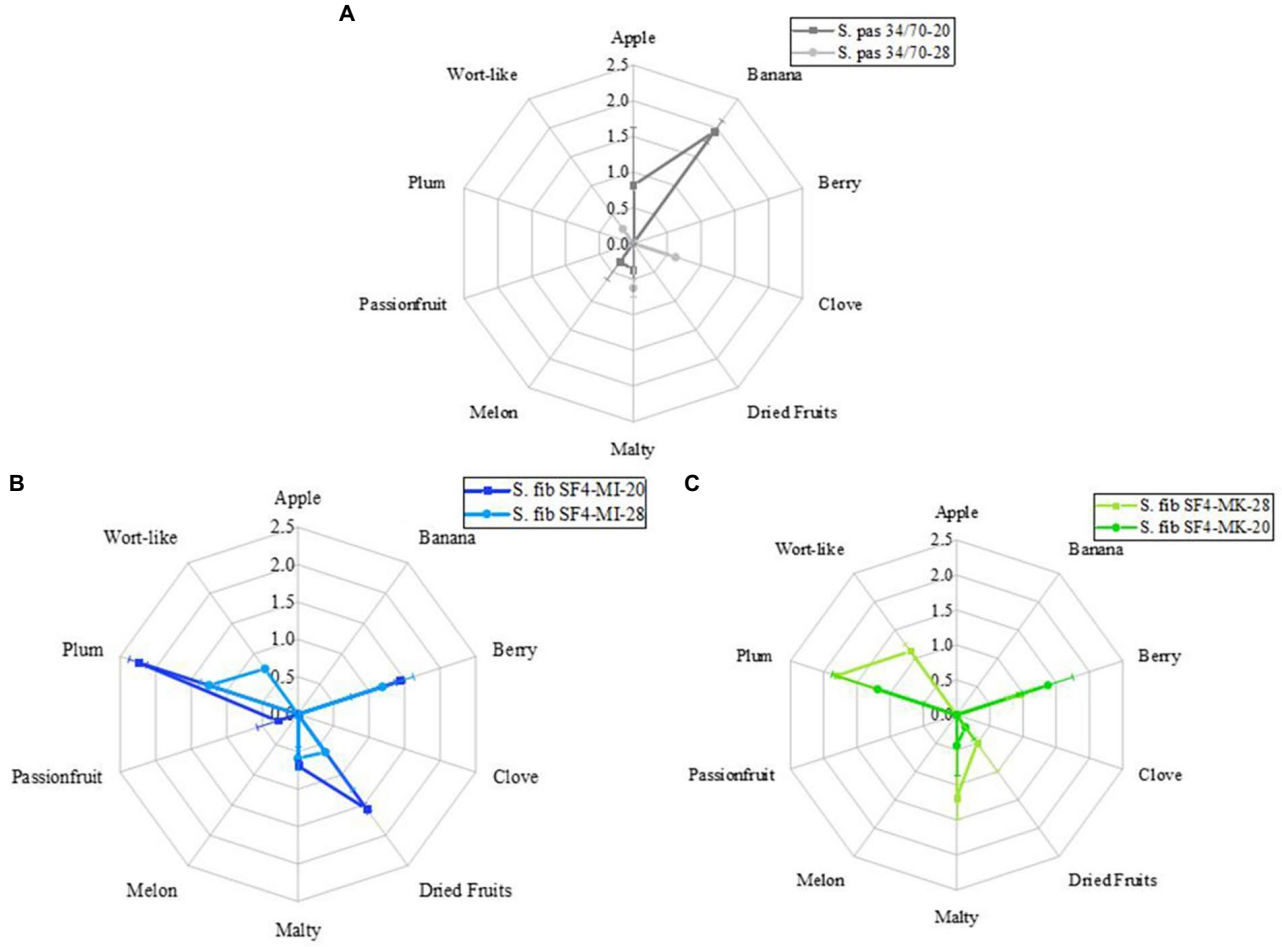
Flavor profiles of the beers fermented with the yeast strain *S. fibuligera* S. fib SF4 as micromanipulated (MI) and mother culture (MK) and the reference yeast strain *S. pastorianus* S. pas 34/70 at 20 and 28°C. Values are shown as means of odor and taste with standard deviations. The number of sensory assessors was *n* = 8.

By comparing the flavor profiles of the beers in Figure 6 (raw data are depicted in Supplementary material Table 5), the different fermentation temperatures at 20°C and 28°C had an obvious impact on the flavors of the beers. The difference is most pronounced in the reference beer, which is visible in the radar plot (Figure 6A).

The reference beer fermented at 20°C exhibited a dominant banana flavor, which was complemented by an apple-like and slightly malty and melon-like flavor. At a fermentation temperature of 28°C, the tasters could no longer detect any significant fruity flavor. Instead, the beer was described as neutral with slightly malty, clove and wort-like flavors. Clove-like flavor is unusual for beers fermented with S. pas 34/70. The clove-like flavor is one of the phenolic off-flavors known in beers fermented with certain *S. cerevisiae* yeast strains. These yeast strains must possess the POF1 gene, which enables the yeast to decarboxylate the phenolic acid ferulic acid to 4-vinyl guaiacol (Meaden and Taylor, 1991; Lodolo et al., 2008). This property is not known for S. pas 34/70. Although utmost care was taken, contamination with *S. cerevisiae* cannot be completely excluded, which could have potentially caused a clove-like flavor in the beer. The wort flavor was also reflected as an off-flavor in the total DLG score and resulted in deductions in the overall score, which is visible in Table 6. While the reference beer fermented at 28°C received the lowest DLG rating of the total of six beers with a score of 4.05 points, the reference beer fermented at 20°C achieved the best score of 4.45 points. In contrast, the flavor profiles of the two beers fermented with the micromanipulated S. fib SF4 yeast culture were more alike as shown in the radar plot (Figure 6B). Both beers exhibited a pronounced plum flavor supported by berry and dried fruit flavors. Moreover, slight malty notes were detected in the beers. In general, however, the flavor expressions were significantly stronger in the beer fermented at 20°C than in the beer fermented at 28°C. Slight wort flavors were noticed in the beer fermented at 28°C, which were not perceived in the beer fermented at 20°C. This slight wort flavor led to a devaluation of the total DLG score here, too, and amounted to 4.22 points. The beer fermented at 20°C scored better at 4.43 and was rated very good. In addition to the aforementioned flavors, the sensory assessors mentioned traces of passion fruit in the beer, which were likely to support the positive overall fruity flavor and were not perceptible in the beer produced at 28°C. The flavor characteristics of the beers fermented with the SF4 mother culture were, similarly to the SF4-MI beers, almost independent of temperature. All four beers produced by using *S. fibuligera* SF4 revealed strong similarities. Accordingly, pronounced plum and berry flavors were detected in the beers fermented with the SF4 mother culture as can be seen in the radar plot (Figure 6C). Additionally, the sensory assessors identified malty and dried fruit flavors, although the dried fruit flavors were less pronounced than in the SF4-MI beers. Wort flavor could again only be perceived in the beer fermented at 28°C, which was thus common to all beers produced at 28°C. Comparing the SF4-MI-20 beer with the SF4-MK-20 beer, it is noticeable that the plum and the dried fruit flavors were significantly more distinct in the beer SF4-MI-20, while the two SF4 beers fermented at 28°C differed significantly less regarding the flavor expressions. Just as with the other two beers, which had slight wort off-flavors, the beer fermented with S. fib SF4-MK-28 was downgraded in the total DLG score and received 4.20 points. The beer produced with S. fib SF4-MK-20 was rated better with 4.35 points. However, due to the lower fruitiness, it may have scored lower than the beer produced with S. fib SF4-MI-20.

**Table 6:** Total DLG score according to the DLG evaluation scheme excluding the quality of bitterness as well as carbonation of the investigated beers fermented with *S. fibuligera* S. fib SF4 yeast cultures and reference yeast strain *S. pastorianus* S. pas 34/70 at 20°C and 28°C.

Yeast culture	Total DLG score
S. pas 34/70-20	4.45
S. fib SF4-MI-20	4.43
S. fib SF4-MK-20	4.35
S. pas 34/70-28	4.05
S. fib SF4-MI-28	4.22
S. fib SF4-MK-28	4.20

## Discussion

Although extensive studies on the extracellular amylase system of the yeast species *S. fibuligera* already exist (Futatsugi et al., 1993; Hostinová, 2002; Chen et al., 2010; Kurtzman and Smith, 2011), it has not yet been investigated whether the yeast possesses transmembrane transporters for maltose and maltotriose with intracellular enzyme systems for their cleavage in addition to extracellular amylases. Therefore, the use of the maltose and maltotriose transport assay with radiolabeled maltose and maltotriose uncovered new insights into the physiology of this yeast species and revealed that both investigated *S. fibuligera* yeast strains possess an active maltose transport system into the cell, besides the extracellular amylase activity. In contrast, an active transport system for maltotriose was not found. Two fundamentally different mechanisms exist for maltose and maltotriose utilization by yeasts (Krogerus et al., 2019). Either the two sugars can be cleaved extracellularly with the help of amylases or they are taken up directly through the membrane into the cell and are hydrolyzed using an intracellular maltase (La Fuente and Sols, 1962; Novak et al., 2004). Transporters such as AGT1 and MTT1 are known to be responsible for the active transport of maltose and maltotriose (Dietvorst et al., 2005; Vidgren and Londesborough, 2012). However, these transporters are able to carry both sugars and their expression is nonspecific (Magalhães et al., 2016). For *S. fibuligera* it can be suspected that another maltose-specific transport system exists. This could involve MALx1-encoded transporters, which are specific to maltose (Rautio and Londesborough, 2003). Nevertheless, for both *S. fibuligera* yeast strains it was irrelevant whether they were propagated on maltose or maltotriose due to the presence of both mechanisms for maltose and maltotriose utilization.

The brewing potential of the two *S. fibuligera* yeast strains was recently demonstrated in two studies by Methner et al. (2019, 2022). Due to the identical sugar transport systems of the two investigated *S. fibuligera* yeast strains, S. fib SF4 and S. fib Lu 27, and the higher fermentation activity of the yeast strain S. fib SF4, the yeast strain S. fib SF4 was selected for all further trials in this study. The filamentous growth of *S. fibuligera* leads to limited usability on an industrial scale. The mycelium does not settle at the bottom of the tank during cold storage, which can lead to difficulties in the clarification of the beer. Since the yeast forms mycelia as well as single cells during the growth phase, single cells were micromanipulated to examine whether they increasingly formed single cells during propagation. Indeed, the micromanipulated single cells developed significantly weaker mycelia than the mother culture during propagation. This occurred independently of the two temperatures selected at 20°C and 28°C. It cannot be conclusively explained yet and would therefore provide potential for further investigation into why significantly less mycelial formation was visible in the micromanipulated cultures. Additionally, future repitching experiments would need to investigate how stable the specific phenotypic trait of the lower mycelial formation of the yeast isolate is in order to reliably apply the benefits of better flocculation and clarification properties on an industrial scale. Investigations with *S. cerevisiae* have shown that so-called FLO genes are responsible for the flocculation behavior, which could possibly also be the case for *S. fibuligera* (Teunissen and Steensma, 1995; Lodolo et al., 2008). It is also known that the flocculation behavior of *S. cerevisiae* can be highly variable, and flocculation behavior could be influenced by selecting specific layers of yeast sediment for reinoculation (Steensels and Verstrepen, 2014). This assumption could be applied to *S. fibuligera* in the context of this study. Another possible suggestion may be that the MIG1 gene was suppressed in the micromanipulated cultures. This gene plays a crucial role in mycelial formation of *S. fibuligera*. Provided it is suppressed, mycelial formation is reduced and cell budding enhanced. Repression of the MIG1 gene in the micromanipulated cultures would also help explain the slightly higher sugar utilization in the corresponding beers in direct comparison to the beers fermented with the mother culture. Repression of the gene enhances the expression of genes such as α-amylase and glucoamylase (Liu et al., 2011).

(GTG)_5_ rep-PCR fingerprints confirmed that identical patterns emerged during capillary gel electrophoresis. (GTG)_5_ rep-PCR typing only confirms a stable state within the investigated target DNA-regions. No genetic changes could be observed in terms of the genetic target spectrum. Nevertheless, a more universal approach like whole genome sequencing could be applied in further studies to reveal a genetic origin of the phenotypic difference or not. (GTG)_5_ rep-PCR typing confirmed that both isolates (clones) still belonged to the same strain origin.

The described morphologies also persisted during the fermentation trials, so micromanipulation might actually be helpful in propagating a culture that exhibits reduced mycelia formation. In this study, the fermentation period was chosen for 20 days in order to bridge a potential prolonged lag phase. Even though such a lag phase was observed in previous studies during the fermentation of wild *Saccharomyces* yeasts (Nikulin et al., 2020; Hutzler et al., 2021) it should not be generally excluded for non-*Saccharomyces* yeasts. While it cannot be completely excluded that the lag phase would have reverted to the active phase at a later stage, based on the extensive stagnation of the fermentation, it could be assumed that there was no delay in fermentation. As shown by the sugar utilization, only about 40–65% of the maltose and 40–50% of the maltotriose were degraded by S. fib SF4 during fermentation across temperatures and regardless of the culture (micromanipulated or mother culture). In contrast, the reference yeast S. pas 34/70 utilized maltose almost completely and maltotriose to nearly 90%. A possible reason for the incomplete utilization of maltose and maltotriose by S. fib SF4 could be an inhibition of the maltose transporter by other substances. Rautio and Londesborough found that, depending on the yeast strain, glucose and maltose transport can be inhibited by each other even though both sugars are carried by different transporters (Rautio and Londesborough, 2003). Since neither glucose nor maltose were fully utilized, this could be a possible explanation. Moreover, it is known that yeast maltose metabolism can be suppressed by glucose metabolism as glucose can repress the transcription of necessary gene loci (such as MIG1) for the corresponding maltose permeases as well as for specific enzymes for the hydrolysis of maltose, which was found in several studies with *Saccharomyces* yeasts (Görts, 1969; Federoff et al., 1983; Klein et al., 1996). Based on the available results, only assumptions can be made at present why the fermentation performance in the brewer’s wort was relatively low. It could be an indication of a possible inhibition mechanism of sugar uptake in brewer’s wort. Furthermore, a transformation of the sugars to organic acids or other fermentation-by-products or a concentration-dependent feedback of fermentation products could have led to the low fermentation performance, too. Based on existing studies, it is known that yeasts can form different organic acids from carbohydrate sources during fermentation instead of producing ethanol. The formation pathways of organic acids are versatile. Acids are basically present in the raw materials of the wort, however, the acid concentrations can be changed by the yeasts during fermentation. Additionally, acids are produced by the yeasts as by-products of their metabolic pathways (Whiting, 1976). Selected yeast species are known for the production of citric acid or pyruvic acid during fermentation (Yalcin et al., 2010; Chidi et al., 2015; Afolabi et al., 2018), while *Lachancea thermotolerans*, besides other yeast species, possess the ability to form high amounts of lactic acid at the expense of ethanol during fermentation (Sgouros et al., 2020; Rodríguez Madrera et al., 2021). Due to the low pH values in the beers, there is a possibility that S. fib SF4 partly converted carbohydrates to organic acids instead of ethanol. Additionally, the glucose and maltose utilization in Figure 5 may appear lower than the actual results as the extracellular amylase system might have cleaved maltotriose and further oligosaccharides. Consequently, relatively higher proportions of maltose and glucose could be present than in the initial wort. Nevertheless, contrary to expectations, the yeast strain S. fib SF4 degraded only small amounts of the original extract as the two beers fermented at 20°C exhibited apparent attenuations of between 14 and 16%, while the two beers fermented at 28°C had apparent attenuations that ranged between approximately 16 and 23%. A higher apparent attenuation was expected since the same yeast strain was close to 47% at a fermentation temperature of 27°C in a previous study by Methner et al. and was thus about twice as high (Methner et al., 2019). Although the results of this study show that different cultures differed by 7% for apparent attenuation despite identical conditions, further experiments need to be conducted in the future to explain why the values can vary significantly. The apparent attenuations of the beers produced with the reference yeast strain S. pas 34/70 were approximately 82%, reaching the final apparent attenuation for lager beers (Narziss et al., 2017). As expected, the apparent attenuation affected the ethanol content in the final beers, so values between 0.83 and 1.20% (*v*/*v*) were obtained for S. fib SF4 at 28°C, while 3.10% (*v*/*v*) ethanol was obtained in the beer from the earlier study by Methner et al. (2019). However, it must be considered that the original gravity of 12.65% was higher than in the present study and so the direct comparison of the apparent attenuations is more accurate. The pH values were similar in a direct comparison of the two studies. While the yeast strain S. fib SF4 reached a drop in pH to 4.30 in the earlier study, the pH values in this study ranged from 4.16–4.43. The low pH values could explain why the α-amylase of S. fib SF4 did not work optimally. According to Xie (Xie et al., 2021), the pH optimum of α-amylase is between 5.0–6.0 and the optimal temperature for this enzyme is between 40–50°C. At the beginning of the fermentation process, the pH of the wort was still within the optimal range at 5.3, which could explain the slightly higher fermentation activity at the beginning of the fermentation process (*cf.* Figure 4). The slightly higher fermentation temperature of 28°C likely accelerated the enzyme activity compared to the fermentation temperature of 20°C and thus led to a comparatively faster fermentation process due to a faster extracellular sugar cleavage. Nevertheless, the optimum temperature of α-amylase was far from being reached and the pH values of the beers dropped during fermentation. Therefore, the extracellular enzyme system most probably worked very slowly. An inhibitory effect of the sugar transport systems could nevertheless be likely and could be specifically tested in further experiments.

The sensory properties of the beers play a crucial role for consumers. As shown in Figure 6, the four experimental beers were depicted next to the two reference beers. In this study, the focus was on the direct comparison between the beers fermented with the micromanipulated and the mother culture. In general, despite the different morphologies of the two cultures, the sensory assessors noted almost identical flavor attributes. These coincided with the flavor characteristics of previous studies regardless of temperature, as the beers exhibited pronounced plum- and berry-like flavors. Dried fruit flavors were also perceived, especially in the beers from the micromanipulated culture. While the reference beer fermented with S. pas 34/70 at 20°C exhibited fruity flavors as known from previous studies (Meier-Dörnberg et al., 2017; Hutzler et al., 2021), the reference beer fermented at 28°C was rather neutral and did not exhibit any conspicuous flavor characteristics. Possibly, at 28°C the volatile flavor compounds were more strongly expelled during unpressurized fermentation than at 20°C. In contrast, previous research revealed that *S. fibuligera* produces desirable floral, fruity, and honey-like flavors in rice wine fermentation and in fermentation in selective media containing different carbohydrate sources (Lee et al., 2018; Xie et al., 2021; Yang et al., 2021), while the flavors in beer were previously described to be plum-, and also berry-like (Methner et al., 2019, 2022). It is interesting to note that the floral and honey flavors, produced from rice wine and selective media fermentation, did not appear in the brewer’s wort fermentation. The beer had a distinctive focus on fruity flavors. This could be directly related to the carbon source, as it significantly influences the volatile and non-volatile metabolites according to a study by Lee et al. (2018). The same study also describes a significant influence of the cultivation time on flavor expressions. Time appeared to be less influential with respect to the yeast species *S. fibuligera* as the same flavor components were retained as for previous studies of between seven, 14 and 20 days (Methner et al., 2019, 2022). The key aroma compounds of a sweet rice alcoholic beverage (sweet rice wine) fermented with *S. fibuligera* have already been studied in detail in a study by Yang et al. (2021), where 43 volatile compounds were identified, with ethyl butanoate, ethyl hexanoate, β-phenylethyl alcohol and 1-octen-3-one with high OAVs being responsible for the key aroma compounds. Also in beer, ethyl butanoate was measured well above the flavor threshold, while ethyl hexanoate remained below the flavor threshold (Methner et al., 2019). Furthermore, there are findings that relate to the genes responsible in the yeast species *S. fibuligera* for encoding alcohol acetyltransferase for volatile acetate ester formation (Moon et al., 2021). Six of these ATF genes were found in *S. fibuligera*, while in direct comparison *S. cerevisiae* possessed only two ATF genes. This could be an explanation for the more complex flavor attributes in foods fermented with *S. fibuligera*. Nevertheless, it remains unclear which key flavor compounds were responsible for the dominant plum flavor in the beer. A possible approach for future research would be to target substances found in plums. Fricker listed 14 main flavor compounds from four different plum varieties out of a total of about 160 identified compounds (Fricker, 1984). These 14 main flavor substances γ-hexalactone, γ-octalactone, γ-decalactone, γ-dodecalactone, linalool, α-terpineol, benzyl alcohol, cis-3-hexenol, trans-2-hexenol, ethyl cinnamate, benzaldehyde, n-hexanoic acid, n-octanoic acid and trans-2-hexenoic acid could serve as indicators. Comparing these to the primary odorants in pale lager beer, none of the 14 flavor compounds are found. Schieberle published 33 primary odorants of pale lager beer in 1991, of which he listed 3-methylbutanol, 2-phenylethanol, 3- and 2-methylbutanoic acid, 4-vinyl-2-methoxyphenol, furaneol, ethyl-butanoate, (*E*)-*β*-damascenone, sotolone, butanoic acid, ethyl hexanoate, and hexanoic acid as the most important ones (Schieberle, 1991). Although the two flavor compounds linalool and terpineol can also be found in beer, they originate from the added hops (Lam et al., 1986), which were not used in this study. The malty impression derived from the wort, which may be more pronounced due to the sterilization process and the associated increased thermal input. As a result of the increased thermal input, various aldehydes, Maillard reaction products and ketones are formed (Siefker and Pollock, 1956; De Schutter et al., 2008; Piornos et al., 2020). Aldehydes, in turn, can cause characteristic wort flavors that are often perceived as off-flavors (Gernat et al., 2020). A slight wort flavor was perceived in the two beers fermented with *S. fibuligera* at 28°C. It cannot be conclusively explained as to why the wort flavor was noticeable as wort-derived aldehyde off-flavors are often degraded by the yeasts during fermentation. This has already been studied for selected non-*Saccharomyces* yeast strains in the context of cold contact fermentations for the production of non-alcoholic beers (Nikulin et al., 2022). Consequently, it could be assumed that the yeast strain S. fib SF4 was also capable of degrading wort-derived aldehyde off-flavors, as the two beers fermented at 20°C did not exhibit this flavor attribute.

## Conclusion

The study clarified whether the yeast species *S. fibuligera* possessed an active transport system for maltose and maltotriose. While an active transport system for maltose was found in the two investigated *S. fibuligera* strains, there was no such system for maltotriose. Thus, it was concluded that in addition to the extracellular amylase system known for *S. fibuligera*, maltose transport also occurred across the cell membrane. Despite the ability of the yeast to hydrolyze and metabolize both maltose and maltotriose, a very slow fermentation was observed compared to the domesticated reference yeast strain *S. pastorianus* TUM 34/70, resulting in low ethanol contents between 0.8–1.2% (*v*/*v*) in the final beers brewed at 10 °P original gravity. Therefore, the yeast species *S. fibuligera* is ideal for producing beer with a low alcohol content, which at the same time has unique flavor properties. To produce regular beers, it would also be conceivable to use *S. fibuligera* in co-culture with domesticated *Saccharomyces* yeasts in future studies, as this could potentially increase flavor complexity. In this study, the brewing potential was also investigated and optimized by using micromanipulation and re-culturing of the yeast strain S. fib SF4 to reduce the characteristic mycelial formation during propagation and fermentation. This was successfully achieved with the help of micromanipulation. The significantly reduced filamentous growth of the yeast can lead to a positive effect on the flocculation and filtration of the beer on an industrial scale. By further comparing the micromanipulated culture to the mother culture, no genetic differences could be detected in the (GTG)_5_ rep-PCR fingerprint. The beers fermented with the two different S. fib SF4 cultures revealed only minimal differences as the beers fermented with the micromanipulated culture displayed slightly higher fermentation activity and exhibited stronger dried fruit flavors in addition to the well-known fruity plum and berry flavors. The flavor of the beers fermented at 20°C was preferred by the tasters over the beer flavor at 28°C fermentation temperature while the beer produced with the micromanipulated S. fib SF4 culture at 20°C scored best.

## Data availability statement

The original contributions presented in the study are included in the article/ Supplementary materials, further inquiries can be directed to the corresponding author.

## Author contributions

YM, FM, and MH conceived the study. YM, FM, and LR performed the experiments. YM and FM analyzed the data. YM and FM wrote the manuscript. YM, FM, LR, MZ, and MH reviewed and edited the manuscript. MZ, FJ, and MH acquired the funding. FJ and MH supervised the study. All authors contributed to the article and approved the submitted version.

## Funding

This research was funded by the BMWK (German Ministry of Economic Affairs and Climate Action), Forschungsvereinigung Wifoe (Association for the Promotion of Science of the German Brewing Industry, Berlin). The IGF Project (grant number AiF 20658 N) of the Wifoe is supported *via* AiF within the program for promoting the Industrial Collective Research (IGF) of the BMWK, based on a decision by the German Bundestag.

## Conflict of interest

Author FM was employed by VTT Technical Research Centre of Finland Ltd.

The remaining authors declare that the research was conducted in the absence of any commercial or financial relationships that could be construed as a potential conflict of interest.

## Publisher’s note

All claims expressed in this article are solely those of the authors and do not necessarily represent those of their affiliated organizations, or those of the publisher, the editors and the reviewers. Any product that may be evaluated in this article, or claim that may be made by its manufacturer, is not guaranteed or endorsed by the publisher.

## References

[ref1] Afolabi F. T. Adeyemo S. M. Balogun H. O. (2018). Fermentation conditions and process optimization of citric acid production by yeasts. Int. J. Biotechnol. 7, 51–63. doi: 10.18488/journal.57.2018.71.51.63

[ref2] Aidoo K. E. Nout M. J. R. Sarkar P. K. (2006). Occurrence and function of yeasts in Asian indigenous fermented foods. FEMS Yeast Res. 6, 30–39. doi: 10.1111/J.1567-1364.2005.00015.X, PMID: 16423068

[ref3] Boundy-Mills K. Stratford M. Miller M. W. (2011). “Chapter 62- Saccharomycodes E.C. Hansen (1904),” in The Yeasts: A taxonomic Study. ed. Kurtzman C. P. (Amsterdam: Elsevier), 747–750. doi: 10.1016/B978-0-444-52149-1.00062-8

[ref4] Budroni M. Zara G. Ciani M. Comitini F. (2017). “*Saccharomyces* and non-*saccharomyces* starter yeasts,” in Brewing Technology. ed. Kanauchi M. (London: Intech Open) doi: 10.5772/intechopen.68792

[ref5] Chen L. Chi Z.-M. Chi Z. Li M. (2010). Amylase production by *Saccharomycopsis Fibuligera* A11 in solid-state fermentation for hydrolysis of cassava starch. Appl. Biochem. Biotechnol. 162, 252–263. doi: 10.1007/S12010-009-8744-3, PMID: 19701612

[ref6] Chidi B. S. Rossouw D. Buica A. S. Bauer F. F. (2015). Determining the impact of industrial wine yeast strains on organic acid production under white and red wine-like fermentation conditions. South Afr. J. Enol. Viticult. 36, 316–327. doi: 10.21548/36-3-965

[ref7] De Schutter D. P. Saison D. Delvaux F. Derdelinckx G. Rock J.-M. Neven H.. (2008). Release and evaporation of volatiles during boiling of Unhopped wort. J. Agric. Food Chem. 56, 5172–5180. doi: 10.1021/Jf800610x, PMID: 18547048

[ref8] Dietvorst J. Londesborough J. Steensma H. Y. (2005). Maltotriose utilization in lager yeast strains: Mtt1 encodes a Maltotriose transporter. Yeast 22, 775–788. doi: 10.1002/Yea.1279, PMID: 16088872

[ref9] Djien K. S. (1972). Tapé Fermentation. Appl. Microbiol. 23, 976–978. doi: 10.1128/Am.23.5.976-978.1972, PMID: 16349926 PMC380483

[ref10] Erdem M. Kesmen Z. Özbekar E. Çetin B. Yetim H. (2016). Application of high-resolution melting analysis for differentiation of spoilage yeasts. J. Microbiol. 54, 618–625. doi: 10.1007/S12275-016-6017-8, PMID: 27572511

[ref11] Esslinger H. M. (2009). Handbook of Brewing: Processes, Technology, Markets. Weinheim, Germany: John Wiley & Sons

[ref12] Federoff H. J. Eccleshall T. R. Marmur J. (1983). Carbon catabolite repression of maltase synthesis in *saccharomyces Carlsbergensis*. J. Bacteriol. 156, 301–307. doi: 10.1128/jb.156.1.301-307.1983, PMID: 6352680 PMC215083

[ref13] Fricker A. (1984). “Die Sensorisch Wirksamen Stoffe In Lebensmitteln,” in Lebensmittel — Mit Allen Sinnen Prüfen! (Springer, Berlin: Heidelberg), 46–117. doi: 10.1007/978-3-642-82325-1_3

[ref14] Futatsugi M. Ogawa T. Fukuda H. (1993). Purification and properties of two forms of Glucoamylase from *Saccharomycopsis Fibuligera*. J. Ferment. Bioeng. 76, 521–523. doi: 10.1016/0922-338x(93)90252-4

[ref15] Gernat D. C. Brouwer E. Ottens M. (2020). Aldehydes as wort off-Flavours in alcohol-free beers—origin and control. Food Bioprocess Technol. 13, 195–216. doi: 10.1007/s11947-019-02374-z

[ref16] Gibson B. R. Storgårds E. Krogerus K. Vidgren V. (2013). Comparative physiology and fermentation performance of Saaz and Frohberg lager yeast strains and the parental species *saccharomyces Eubayanus*. Yeast 30, 255–266. doi: 10.1002/Yea.2960, PMID: 23695993

[ref17] Görts C. P. M. (1969). Effect of glucose on the activity and the kinetics of the maltose-uptake system and of Α-glucosidase in *saccharomyces cerevisiae*. Biochim. Biophys. Acta Gen. Subj. 184, 299–305. doi: 10.1016/0304-4165(69)90032-4, PMID: 5809715

[ref18] Hildebrandt G. Schneider-Haeder B. (2009). Sensorische Analyse: Methodenüberblick Und Einsatzbereiche–Dlg-Sensorik, 2/2009, Www.Dlg.Org

[ref19] Hostinová E. (2002). Amylolytic enzymes produced by the yeast *Saccharomycopsis Fibuligera*. Biologia 57, 247–252.

[ref20] Hostinová E. Gašperík J. (2010). Yeast Glucoamylases. Mol. Genet. Struct. Charact. 65, 559–568. doi: 10.2478/S11756-010-0077-8

[ref21] Hutzler M. Michel M. Kunz O. Kuusisto T. Magalhães F. Krogerus K.. (2021). Unique brewing-relevant properties of a strain of *saccharomyces Jurei* isolated from ash (Fraxinus Excelsior). Front. Microbiol. 12:645271. doi: 10.3389/Fmicb.2021.645271, PMID: 33868204 PMC8044551

[ref22] Janeček Š. Baláž Š. (1992). Α-amylases and approaches leading to their enhanced stability. FEBS Lett. 304, 1–3. doi: 10.1016/0014-5793(92)80575-2, PMID: 1618293

[ref23] Klein C. J. Olsson L. Rønnow B. Mikkelsen J. D. Nielsen J. (1996). Alleviation of glucose repression of maltose metabolism by Mig1 disruption in *saccharomyces cerevisiae*. Appl. Environ. Microbiol. 62, 4441–4449. doi: 10.1128/Aem.62.12.4441-4449.1996, PMID: 8953715 PMC168270

[ref24] Kreger-Van Rij N.J.W. (1984). The Yeasts: a Taxonomic Stud*y*. Amsterdam: Elsevier Science Publishers B. V.

[ref25] Krogerus K. Magalhães F. Kuivanen J. Gibson B. (2019). A deletion in the Sta1 promoter determines Maltotriose and starch utilization in Sta1+ *saccharomyces cerevisiae* strains. Appl. Microbiol. Biotechnol. 103, 7597–7615. doi: 10.1007/S00253-019-10021-Y, PMID: 31346683 PMC6719335

[ref26] Kurtzman C. P. Smith M. T. (2011). “Chapter 63- Saccharomycopsis Schiönning (1903): *Saccharomycopsis Fibuligera* (Lindner) Klöcker (1924),” in The Yeasts: A Taxonomic Study. ed. Kurtzman C. P. (Amsterdam: Elsevier), 755–756.

[ref27] La Fuente G. Sols A. (1962). Transport of sugars in yeasts. Biochim. Biophys. Acta 56, 49–62. doi: 10.1016/0006-3002(62)90526-7 13884257

[ref28] Lam K. C. Foster R. T. Deinzer M. L. (1986). Aging of hops and their contribution to beer flavor. J. Agric. Food Chem. 34, 763–770. doi: 10.1021/jf00070a043

[ref29] Lee S. M. Jung J. H. Seo J.-A. Kim Y.-S. (2018). Bioformation of volatile and nonvolatile metabolites by *Saccharomycopsis Fibuligera* Kjj81 cultivated under different conditions-carbon sources and cultivation times. Molecules 23:2762. doi: 10.3390/Molecules23112762, PMID: 30366381 PMC6278445

[ref30] Lindner P. (1907). *Endomyces Fibuliger* N. Sp. Ein Neuer Gärungspilz Und Erzeuger Der Sog. Kreidekrankheit Des Brotes. Wochenschrift Für Brauerei 24, 469–474.

[ref31] Liu G.-L. Wang D.-S. Wang L.-F. Zhao S.-F. Chi Z.-M. (2011). Mig1 is involved in mycelial formation and expression of the genes encoding extracellular enzymes in *Saccharomycopsis Fibuligera* A11. Fungal Genet. Biol. 48, 904–913. doi: 10.1016/j.fgb.2011.04.008, PMID: 21558012

[ref32] Lodolo E. J. Kock J. L. F. Axcell B. C. Brooks M. (2008). The yeast *saccharomyces cerevisiae*-the Main character in beer brewing. FEMS Yeast Res. 8, 1018–1036. doi: 10.1111/J.1567-1364.2008.00433.X, PMID: 18795959

[ref33] Lucero P. Peñalver E. Moreno E. Lagunas R. (1997). Moderate concentrations of ethanol inhibit endocytosis of the yeast maltose transporter. Appl. Environ. Microbiol. 63, 3831–3836. doi: 10.1128/Aem.63.10.3831-3836.1997, PMID: 9327546 PMC168692

[ref34] Magalhães F. Vidgren V. Ruohonen L. Gibson B. (2016). Maltose and Maltotriose utilisation by group I strains of the hybrid lager yeast *saccharomyces Pastorianus*. FEMS Yeast Res. 16:fow053. doi: 10.1093/Femsyr/Fow053, PMID: 27364826 PMC5815069

[ref35] Meaden P. G. Taylor N. R. (1991). Cloning of a yeast gene which causes phenolic off-Flavours in beer. J. Inst. Brew. 97, 353–357. doi: 10.1002/J.2050-0416.1991.Tb01075.X

[ref36] Meier-Dörnberg T. Hutzler M. Michel M. Methner F.-J. Jacob F. (2017). The importance of a comparative characterization of *saccharomyces cerevisiae* and *saccharomyces Pastorianus* strains for brewing. Fermentation 3:41. doi: 10.3390/Fermentation3030041

[ref37] Methner Y. Hutzler M. Matoulková D. Jacob F. Michel M. (2019). Screening for the brewing ability of different non-*saccharomyces* yeasts. Fermentation 5:101. doi: 10.3390/Fermentation5040101

[ref38] Methner Y. Hutzler M. Zarnkow M. Prowald A. Endres F. Jacob F. (2022). Investigation of non-*saccharomyces* yeast strains for their suitability for the production of non-alcoholic beers with novel flavor profiles. J. Am. Soc. Brew Chem. 1–15, 1–15. doi: 10.1080/03610470.2021.2012747

[ref39] Meyer W. Mitchell T. G. Freedman E. Z. Vilgalys R. (1993). Hybridization probes for conventional Dna fingerprinting used as single primers in the polymerase chain Reaction to distinguish strains of *Cryptococcus Neoformans*. J. Clin. Microbiol. 31, 2274–2280. doi: 10.1128/Jcm.31.9.2274-2280.1993, PMID: 8408543 PMC265746

[ref40] Michel M. Kopecká J. Meier-Dörnberg T. Zarnkow M. Jacob F. Hutzler M. (2016). Screening for new brewing yeasts in the non-*saccharomyces* sector with *Torulaspora Delbrueckii* as model. Yeast 33, 129–144. doi: 10.1002/Yea.3146, PMID: 26647111

[ref41] Moon H. Y. Kim H. J. Kim K. S. Yoo S. J. Lee D. W. Shin H. J.. (2021). Molecular characterization of the *Saccharomycopsis Fibuligera* Atf genes, encoding alcohol acetyltransferase for volatile acetate Ester formation. J. Microbiol. 59, 598–608. doi: 10.1007/S12275-021-1159-8, PMID: 34052992

[ref42] Narziss L. Back W. Gastl M. Zarnkow M. (2017). Abriss Der Bierbrauerei. Newark: John Wiley & Sons Incorporated

[ref43] Narziß L. Back W. Miedaner H. Lustig S. (1999). Untersuchung Zur Beeinflussung Der Geschmacksstabilität Durch Variation Technologischer Parameter Bei Der Bierherstellung. Monatsschrift Für Brauwissenschaft 1999, 192–206.

[ref44] Nga B. H. Yip C. W. Koh S. I. Chiu L. L. (1995). Variation of electrophoretic karyotypes in genetically different strains of *Saccharomycopsis Fibuligera* and *Yarrowia Lipolytica*. Microbiology 141, 705–711. doi: 10.1099/13500872-141-3-705

[ref45] Nikulin J. Aisala H. Gibson B. (2022). Production of non-alcoholic beer via cold contact fermentation with *Torulaspora Delbrueckii*. J. Inst. Brew. 128, 28–35. doi: 10.1002/Jib.681

[ref46] Nikulin J. Eerikäinen R. Hutzler M. Gibson B. (2020). Brewing characteristics of the Maltotriose-positive yeast *Zygotorulaspora Florentina* isolated from oak. Beverages 6:58. doi: 10.3390/Beverages6040058

[ref47] Novak S. Zechner-Krpan V. Marić V. (2004). Regulation of maltose transport and metabolism in *saccharomyces cerevisiae*. Food Technol. Biotechnol. 42, 213–218.

[ref48] Pandey A. (1995). Glucoamylase research: an overview. Starch/Stärke 47, 439–445. doi: 10.1002/Star.19950471108

[ref49] Piornos J. A. Balagiannis D. P. Methven L. Koussissi E. Brouwer E. Parker J. K. (2020). Elucidating the odor-active aroma compounds in alcohol-free beer and their contribution to the Worty flavor. J. Agric. Food Chem. 68, 10088–10096. doi: 10.1021/acs.jafc.0c03902, PMID: 32799537 PMC7499417

[ref50] Rautio J. Londesborough J. (2003). Maltose transport by Brewer's yeasts in Brewer's wort. J. Inst. Brew. 109, 251–261. doi: 10.1002/J.2050-0416.2003.Tb00166.X

[ref51] Riedl R. Dünzer N. Michel M. Jacob F. Hutzler M. (2019). Beer enemy number one: genetic diversity, physiology and biofilm formation of *lactobacillus brevis*. J. Inst. Brew. 125, 250–260. doi: 10.1002/Jib.553

[ref52] Rodríguez Madrera R. Pando Bedriñana R. Suárez Valles B. (2021). Evaluation of indigenous non-*saccharomyces* cider yeasts for use in brewing. Eur. Food Res. Technol. 247, 819–828. doi: 10.1007/S00217-020-03665-Y

[ref53] Schieberle P. (1991). Primary odorants of pale lager beer. Eur. Food Res. Technol. 193, 558–565. doi: 10.1007/BF01190873

[ref54] Sgouros G. Mallouchos A. Filippousi M.-E. Banilas G. Nisiotou A. (2020). Molecular characterization and enological potential of a high lactic acid-producing *Lachancea Thermotolerans* vineyard strain. Foods 9:595. doi: 10.3390/Foods9050595, PMID: 32392718 PMC7278797

[ref55] Siefker J. A. Pollock G. E. (1956). Melanoidins in the brewing processes. I. Formation of aldehydes during wort boiling. Proceedings. Annu. Meet. Am. Soc. Brew. Chem. 14, 5–12. doi: 10.1080/00960845.1956.12006472

[ref56] Srithamma L. (2009). Defining and optimization of Satho production technology. Dissertation. Technische Universität München, Fakultät Wissenschaftszentrum Weihenstephan.

[ref57] Steensels J. Verstrepen K. J. (2014). Taming wild yeast: potential of conventional and nonconventional yeasts in industrial fermentations. Annu. Rev. Microbiol. 68, 61–80. doi: 10.1146/Annurev-Micro-091213-113025, PMID: 24773331

[ref58] Teunissen A. W. Steensma H. Y. (1995). Review: the dominant flocculation genes of *saccharomyces cerevisiae* constitute a new Subtelomeric gene family. Yeast 11, 1001–1013. doi: 10.1002/Yea.320111102, PMID: 7502576

[ref59] Tiwari S. P. Srivastava R. Singh C. S. Shukla K. Singh R. K. Singh P.. (2015). Amylases: an overview with special reference to alpha amylase. J. Glob. Biosci. 4, 1886–1901.

[ref60] Versalovic J. Schneider M. De Brujin F. J. Lupski J. R. (1994). Genomic fingerprinting of bacteria using repetitive sequence-based polymerase chain reaction. Meth. Mol. Biol. 5, 25–40.

[ref61] Vidgren V. Londesborough J. (2012). Characterization of the *saccharomyces Bayanus*-type Agt1 transporter of lager yeast. J. Inst. Brew. 118, 148–151. doi: 10.1002/Jib.22

[ref62] Whiting G. C. (1976). Organic acid metabolism of yeasts during fermentation of alcoholic beverages - a review. J. Inst. Brew. 82, 84–92. doi: 10.1002/J.2050-0416.1976.Tb03731.X

[ref63] Wickerham L. J. Lockwood L. B. Pettijohn O. G. Ward G. E. (1944). Starch hydrolysis and fermentation by the yeast *Endomycopsis Fibuliger*. J. Bacteriol. 48, 413–427. doi: 10.1128/Jb.48.4.413-427.1944, PMID: 16560847 PMC373986

[ref64] Xie Z.-B. Zhang K.-Z. Kang Z.-H. Yang J.-G. (2021). *Saccharomycopsis Fibuligera* in liquor production: a review. Eur. Food Res. Technol. 247, 1569–1577. doi: 10.1007/S00217-021-03743-9

[ref65] Yalcin S. K. Bozdemir M. T. Ozbas Z. Y. (2010). Citric acid production by yeasts: fermentation conditions, process optimization and strain improvement. Curr. Res. Technol. Educ. Topics Appl. Microbiol. Microbi. Biotechnol. 9, 1374–1382.

[ref66] Yang Y. Zhong H. Yang T. Lan C. Zhu H. (2021). Characterization of the key aroma compounds of a sweet Rice alcoholic beverage fermented with *Saccharomycopsis Fibuligera*. J. Food Sci. Technol. 58, 3752–3764. doi: 10.1007/S13197-020-04833-4, PMID: 34471299 PMC8357862

